# An acceleration of microwave-assisted transesterification of palm oil-based methyl ester into trimethylolpropane ester

**DOI:** 10.1038/s41598-020-76775-y

**Published:** 2020-11-12

**Authors:** Nur Atiqah Mohamad Aziz, Robiah Yunus, Hamidah Abd Hamid, Alsultan Abdul Kareem Ghassan, Rozita Omar, Umer Rashid, Zulkifly Abbas

**Affiliations:** 1 Department of Chemical and Environmental Engineering, Engineering Faculty, Universiti Putra Malaysia, 43400 UPM Serdang, Selangor Malaysia; 2 Institute of Plantation Studies, Universiti Putra Malaysia, 43400 UPM Serdang, Selangor Malaysia; 3 Green Chemistry and Sustainable Engineering Technology Cluster, Centre for Research and Innovation, Universiti Kuala Lumpur, Branch Campus Malaysian Institute of Chemical and Bioengineering Technology, Lot 1988, Taboh Naning, 78000 Alor Gajah, Melaka Malaysia; 4 Institute of Advanced Technology, Universiti Putra Malaysia, 43400 UPM Serdang, Selangor Malaysia; 5 Department of Physics, Faculty of Science, Universiti Putra Malaysia, 43400 UPM Serdang, Selangor Malaysia

**Keywords:** Microwave chemistry, Chemical engineering, Chemical engineering

## Abstract

Microwave-assisted synthesis is known to accelerate the transesterification process and address the issues associated with the conventional thermal process, such as the processing time and the energy input requirement. Herein, the effect of microwave irradiation on the transesterification of palm oil methyl ester (PME) with trimethylolpropane (TMP) was evaluated. The reaction system was investigated through five process parameters, which were reaction temperature, catalyst, time, molar ratio of TMP to PME and vacuum pressure. The yield of TMP triester at 66.9 wt.% and undesirable fatty soap at 17.4% were obtained at 130 °C, 10 mbar, sodium methoxide solution at 0.6 wt.%, 10 min reaction time and molar ratio of TMP to PME at 1:4. The transesterification of palm oil-based methyl ester to trimethylolpropane ester was 3.1 folds faster in the presence of microwave irradiation. The total energy requirement was markedly reduced as compared to the conventional heating method. The findings indicate that microwave-assisted transesterification could probably be an answer to the quest for a cheaper biodegradable biolubricant.

## Introduction

A lubricant market in 2017 was estimated to be US$ 117.4 billion reaching US$ 146.3 billion by the year 2024, with a compound annual growth rate (CAGR) slightly above 3% from 2017 to 2024^1^. From 2017 to 2024, industrial machinery and equipment are expected to be the fastest-growing applications with 4.5% of increment and the petroleum-based lubricant took around 40% of lubricant market^2^. Biolubricant consumption in Germany reached 4% of 1.05 million tons of the total lubricants in 2005 and increased quickly, exceeding 3.5% per year^3^. This urgency is due to the rising concern towards environmental issues contributed by a traditional material, which is mineral oil. Biolubricant is also a safer candidate for environmental issues such as oil spillage and depletion of fossil oil^2,4,5^.

Centuries ago, vegetable oil was an option for lubricating oil, instead of the animal fat for it is safe for the environment, not toxic, easily degradable, replenishable source and abundantly available^6–9^. The high molecular weight of the vegetable oil results in low vaporization of the oil. However, the long chain and unsaturated carbon bonds, specifically the β-carbon, possessed by vegetable oil is susceptible to oxidation, thus leads to low oxidative and thermal stability^10–12^. Therefore, tailoring the chemical structure of the vegetable to synthetic ester is the most promising way to enhance the oil’s properties such as viscosity index, low temperature performance, thermal stability and oxidative stability^7,8,13–16^. Malaysia as a crop plant producer, has various palm-based products such as palm oil methyl ester (PME), which can be utilized to produce new derivatives. The Malaysian government also encourages and supports the private companies to develop biolubricants for automotive and industrial users^17^. The commonly used polyhydric alcohols to produce polyol ester are neopentylglycol (NPG), trimethylolpropane (TMP) and pentaerythritol (PE). Indeed, TMP has reasonable price and easy to handle, while NPG and PE may associate with sublimation problem^18,19^. Therefore, we are interested to explore the process of trimethylolpropane ester (TMPE) production from TMP and PME with a heating intensification via microwave heating.

Transesterification is the most common route to produce esters for lubricant base oil via thermal heating with the presence of catalyst under prescribed operating condition^20,21^. Transesterification of PME and trimethylolpropane consists of three consecutive and reversible reactions with intermediates and by-product as listed in the equation below. Three moles of PME reacts with one mole of TMP to produce three moles of methanol (as by-product) and one mole of TMP triesters (TMPTE).

1 $${\text{TMP }} + {\text{ PME }} \leftrightarrow {\text{ TMP monoester }} + {\text{ methanol}}$$

2 $${\text{TMP monoester }} + {\text{ PME }} \leftrightarrow {\text{ TMP diesters }} + {\text{ methanol}}$$

3 $${\text{TMP diesters }} + {\text{ PME }} \leftrightarrow {\text{ TMP triesters }} + {\text{ methanol}}$$

For the past years, catalytic and enzymatic transesterification have been conducted, such as enzyme^12,22,23^, acid^24,25^ and alkaline^13,26–28^. The reaction using enzyme catalyst took long time, up to 7.4 h at a mild temperature of 70 °C in accord to the ability of enzyme sustainability^29^. Acid catalyst is corrosive for the reactor and toxic in nature^30^. The use of alkaline based catalysts under a vacuum condition has remarkably shortened the reaction time, but the competing saponification reaction of the alkaline catalyst reduced the product yield^26,31,32^. The transesterification of PME and TMP catalysed by 0.3% w/w of calcium methoxide resulted in 9.2 mg dried soap/g after 8 h, while the use of 0.9% w/w of sodium methoxide catalyst led to 46 mg dried soap/g after 1 h. Both conditions achieved 98% of TMPTE^26,31^. The conventional batch stirred tank reactor that used 0.28% w/w sodium methoxide solution in methanol, led to 85.6 mg fatty soap/g with the TMPTE yield of 80.1% also at 1 h of reaction time^31^. The long reaction time has attribute to the accumulation of fatty soap produced.

Microwave-assisted biodiesel production via esterification or transesterification between selected oils and short chain alcohols, such as methanol and ethanol, took a reaction time in the range of 30 s to 15 min with 93% yield of product^33–35^. Moreover, a minimal usage of alkaline catalyst during the microwave-assisted reactions in these studies has suppressed the saponification product. Unfortunately, a lack of studies focused on the transesterification of branched alcohol; TMP and PME at vacuum condition via a microwave pulsed width modulation (microwave PWM) heating mechanism.

Moreover, the side reaction that comes along with hydrolysis reaction in the system, for instance, saponification reaction is scantly available in the literature. Thus, the aim of this study is to investigate the potential of a vacuum operated microwave reactor for the synthesis of TMPE from TMP and PME as a lubricant base oil by suppressing the saponification reaction. This is probably an answer to the quest for a less fatty soap generation in the biolubricant production. The performance of the reaction was then compared with a conventional synthesis method.

## Results and discussion

### Effect of reaction temperature

Microwave heating works based on the principle governed by the polarity of each component that rapidly generates heat at its molecular level^36–38^. In the current study, the microwave heat was rapidly released within the reaction media as a result from the rotation of molecular dipoles of alcohol (TMP) and PME. On the contrary, the previous conventional reaction involved a thermal heat reflux from the surface of the reactor towards the centre of the reaction mixture, where the reaction took a longer time due to the heat loss to surrounding^39,40^.

**Figure 1:**
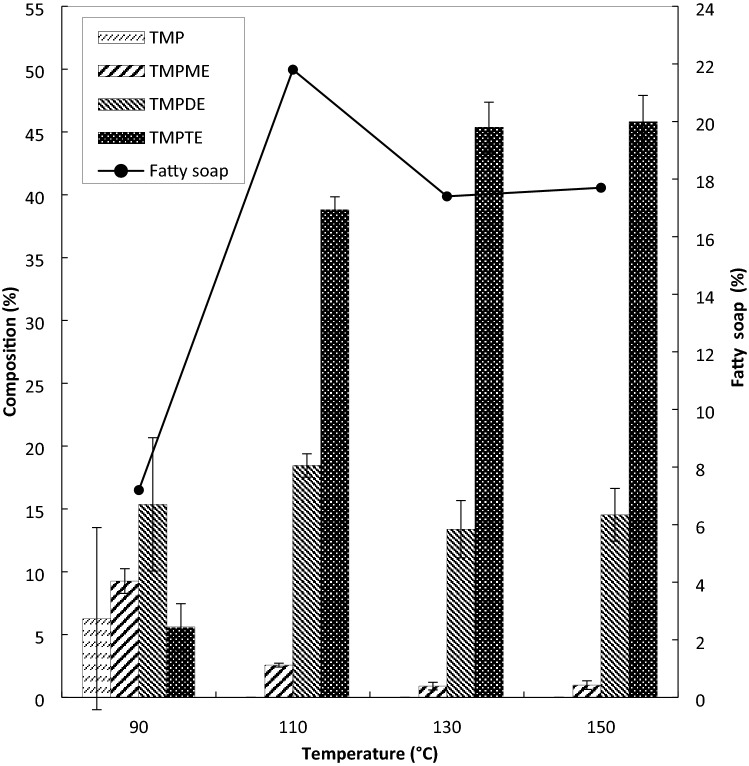
Effect of temperature on composition of TMP esters under pressure of 10 mbar, at 0.6 wt% sodium methoxide catalyst, molar ratio of TMP:PME at 1:4 and reaction time at 10 min.

The reaction was conducted at 90, 110, 130 and 150 °C by a pulsed-modulation microwave reactor with a tolerance of ± 2 °C. Other operating parameters were maintained constant at 1 wt.% amount of catalyst based on the total weight of reactants, 10 min of reaction time, molar ratio of TMP to PME at 1:4 and pressure under 10 mbar. Based on Fig. 1, an increasing trend of TMPTE composition at increasing temperature was observed.

The highest composition of TMPTE at 45.36 wt.% was obtained at 130 °C, the intermediate products of trimethylolpropane diesters (TMPDE) and trimethylolpropane monoester (TMPME) at 13.4 wt.% and 0.9 wt.%, respectively. The difference in TMPTE composition at temperatures of 130 and 150 °C was marginal, with 45.4 wt.% and 45.8 wt.% of TMPTE, respectively. The transesterification reaction at 90 °C was not effective since the reaction was incomplete with a huge amount of unreacted trimethylolpropane in the product. However, a low amount of fatty soap was also observed. This is due to lower occurrence of the side reaction at low temperature. At higher temperatures, the transesterification between TMP and PME was completed because no TMP was left in the product. As the temperature increases, the dielectric constant also increases^36^. The increasing value of the dielectric constant increases the kinetic energy and reduces the viscosity, thus resulting in an easier rotation and faster response to the changes in electrical fields^36,41^.

The composition of TMPDE at 150 °C was slightly high, which may be due to an inevitable reverse reaction of TMPTE to TMPDE. Although high temperature improves the homogeneity of the reacting mixture, after a certain limit, particularly at 150 °C, PME may have vaporised and hence the excess reactant is reduced, which results in the backward reaction^42^. Generally, the reaction is faster at higher temperature. Once the soap was produced, it continued to accumulate until the end of reaction. Therefore, by the end of reaction at 150 °C, 17.7% of fatty soap was produced. Meanwhile, at 110 °C, the highest amount of fatty soap (21.8% of fatty soap) was recorded and a low yield of TMPTE was observed. The result infers that the maximum rate of saponification may happen at 110 °C. The fatty soap production at 110 and 130 °C were 21.8% and about 17.4%, respectively. Higher temperature promotes water removal from the system. Hence, the decrement of free fatty acids generated from the hydrolysis reaction of esters may contribute to lower fatty soap formation^31^. Therefore, with 45.4% yield of TMPTE, 130 °C was selected as the optimum temperature. The standard deviation (SD) of each product, TMPME, TMPDE, and TMPTE are between 0.15–0.99, 0.93–5.3, and 1.04–2.1, respectively.

### Effect of catalyst

The alkaline sodium methoxide catalyst was selected in this study due to the small amount required for a reaction under mild conditions compared to an acid catalyst^13,14,30,42^. In the present study, the use of sodium methoxide has induced an ionic conduction heating mechanism. The movement of ions that in a mixture or solution, occurs under the influence of an electrical field^36,43^. This condition results in a higher collision rate, a higher conversion of kinetic energy to heat, and hence, an intensification of the energy produced. Ionic conductivity governs the energy dissipation instead of dipolar rotation at the initial stage of reaction. The detailed mechanism is shown in the previous studies^44^.

**Figure 2:**
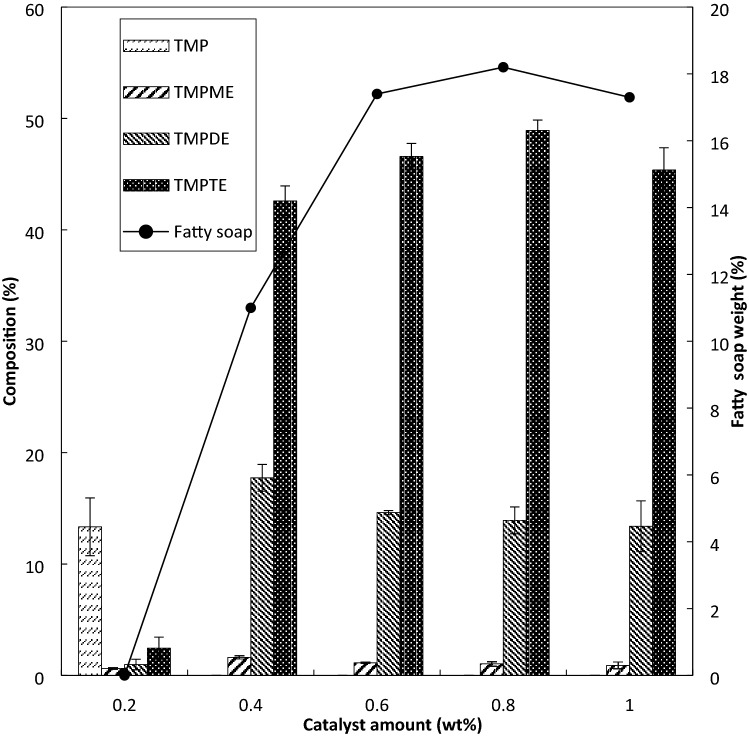
Effect of catalyst amount on esters composition (at pressure 10 mbar, temperature 130 °C, molar ratio 1:4, time 10 min).

The effects of the catalyst were investigated at 0.2, 0.4, 0.6, 0.8 and 1.0 wt.%. Other factors were kept constant at 130 °C, 10 mbar, reaction time of 10 min and molar ratio of TMP to PME at 1:4. The effect of catalyst amount on TMPTE composition was insignificant as shown in Fig. 2. At 0.6 wt.% of sodium methoxide, the composition of TMPME, TMPDE, and TMPTE were 1.13, 14.6 and 46.6 wt.%, respectively. Only a small difference of TMPTE composition was observed for reaction at 0.6 wt.% catalyst (46.6 wt.% TMPTE) as compared to 0.8 wt.% catalyst (48.9 wt.% TMPTE). The standard deviation (SD) of each product, TMPME, TMPDE, and TMPTE are between 0.03–0.31, 0.18–2.28, and 0.94–2.0, respectively.

It was observed that 0.2 wt.% of the catalyst was not enough to push the reaction forward, as shown in Fig. 2. When only a small accumulation of methanol vapour by-product is formed, the vapour may have been trapped in the liquid reaction mixture. For this reason, the methanol removal from the reaction system becomes difficult and the transesterification to produce TMPTE is hindered. For 0.4 wt.% catalyst, the TMPTE composition was 42.6 wt.% but a higher TMPDE composition of 18 wt.% was obtained, compared to 14.6 wt.% of TMPDE when using 0.6 wt.% catalyst. Yunus et al.^45^ and Hamid et al.^14^ reported small TMPDE compositions of 1 wt.% and 7 wt.%, in their final products. Furthermore, the authors reported that a small composition of respectively TMPDE has an added value to the viscosity of the base oil product. From this study, despite of a low fatty soap amount of 11% with a catalyst at 0.4 wt.%, a trace amount of TMP in several experimental runs indicates that the reactions may not be 100% complete. Therefore, the catalyst amount of 0.6 wt.% sodium methoxide was chosen as the best condition because the reaction has reached completion with the amount of fatty soap of 17.4%.

The moisture that probably exists together with TMP, PME, or the catalyst itself, may cause hydrolysis of PME to produce fatty acids. Fatty acids that react irreversibly with sodium methoxide to generate fatty soap, reduce the final product^13,30,42^. The increasing trends in fatty soap by-product was observed for the reactions that used catalyst from 0.4 to 0.8 wt.%. Basically, the fatty soap production was proportional to the catalyst amount. A slight decrease of fatty soap was noticed at 1 wt.% catalyst. This condition may be caused when the catalyst has reached its full capacity and any further addition of catalyst would not expedite the main or side reactions^31^.

In the conducted experiments, methanol was a by-product of transesterification between PME and TMP. Methanol was also present in the catalyst, which was commercially available as 70% in the sodium methoxide solution. Thus, for 0.6 wt.% of catalyst used, only 0.18 wt.% is pure sodium methoxide. Methanol is known for polar molecules, which absorb microwaves better than PME. The high polarity of methanol, with a dielectric constant of 23.03 ± 0.04 and a loss factor of 11.56 ± 0.13 at 30 °C, proves that methanol can be heated rapidly by microwave and then transfer the heat to the surrounding mixture in the reactor. The hot spot that may occur in the reactor is probably because of the concentration of heat due to the presence of methanol. This hot spot also helps in providing rapid heating to the reactant mixture^46^. Table 1 shows the properties of the dielectric constant, ε’, and the loss factor, ε”, for the starting substances that have an important relation to the microwave heating. PME has a high value of dielectric constant, ε’, and loss factor, ε”, of 3.39 and 0.25, respectively, compared to the low values of TMP’s dielectric constant and loss factor, which are 1.78 and 0.04, respectively. This postulates that the heating process was started with PME and then transferred to the mixture of reactants for the transesterification to occur.

**Table 1:** Dielectric constant and loss factor of reactants.

At 2.45 GHz	Measured value	
Substances	Dielectric constant, ε’	Loss factor, ε”
Trimethylolpropane	1.7759	0.0394
Palm oil methyl ester	3.387	0.2505
Water	77.022	9.5395
Air	0.9922	0.0079
Sodium methoxide	2.2203	0.1599

### Effect of reaction time

**Figure 3:**
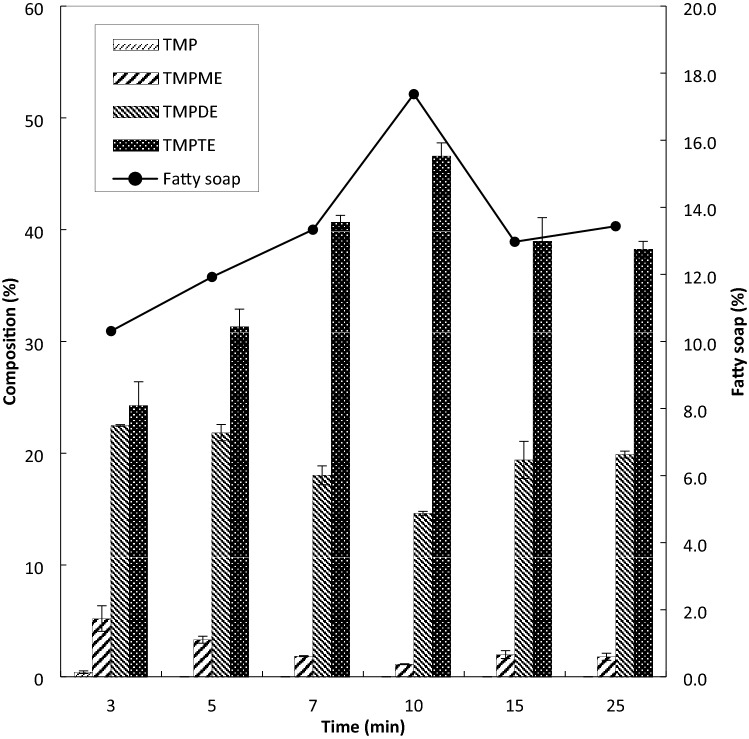
Effect of reaction time on esters composition (at temperature 130 °C, pressure 10 mbar, catalyst 0.6 wt%, molar ratio 1:4).

The application of the electromagnetic wave heating method saves a huge amount of time, especially during the pre-heating of the sample to the prescribed temperature. Using this method, the sample required only 3 min to reach 130 °C as compared to the conventional heating method, which requires around 40 to 60 min to reach the same temperature. In Fig. 3, the effect of reaction time on the ester composition was observed. The reaction was conducted for 3, 5, 7, 10, 15 and 25 min. Other factors were kept constant at 130 °C, 0.6 wt.% catalyst amount, molar ratio of TMP to PME at 1:4, and pressure of 10 mbar. It was observed that 3 min of reaction was insufficient to complete the reaction as a trace of TMP was found in the product. When the duration was prolonged to 10 min of reaction, TMPME, TMPDE and TMPTE compositions were obtained at 1.1, 14.6 and 46.6 wt.%, respectively. The standard deviation (SD) of each product, TMPME, TMPDE, and TMPTE are between 0.04–1.14, 0.08–1.66, and 0.61–2.12, respectively.

A decrease in TMPTE composition was observed after 10 min. The amount of reactant, such as TMP, was insufficient to suppress the backward reaction steps of transesterification. Hence, 10 min is considered as the optimum reaction time because of the highest TMPTE and the lowest TMPME and TMPDE compositions recorded at this condition. The fatty soap content was also low when the reaction time was short, for example, 10.3% of fatty soap at 3 min. This is because prolonging the reaction time promotes soap generation with 13% and 13.4% of fatty soap at 15 and 25 min, respectively.

Interestingly, the result was contrary to the studies conducted by various researchers that reported yield of ester was above 90% at 30 s to 15 min of transesterification and esterification reactions^34,35,47^. The current result could be due to the polarity factor of the starting materials. The previous studies used high polarity of methanol as a starting material in biodiesel production, which increases the vibration frequency of the atoms present in the reaction interfaces with palm fatty acid distillate (PFAD). The polar component is more attracted to microwaves as the dipole–dipole forces respond to the electromagnetic field, which dissipate the energy absorbed from the electromagnetic field^48,49^.

### Effect of molar ratio

**Figure 4:**
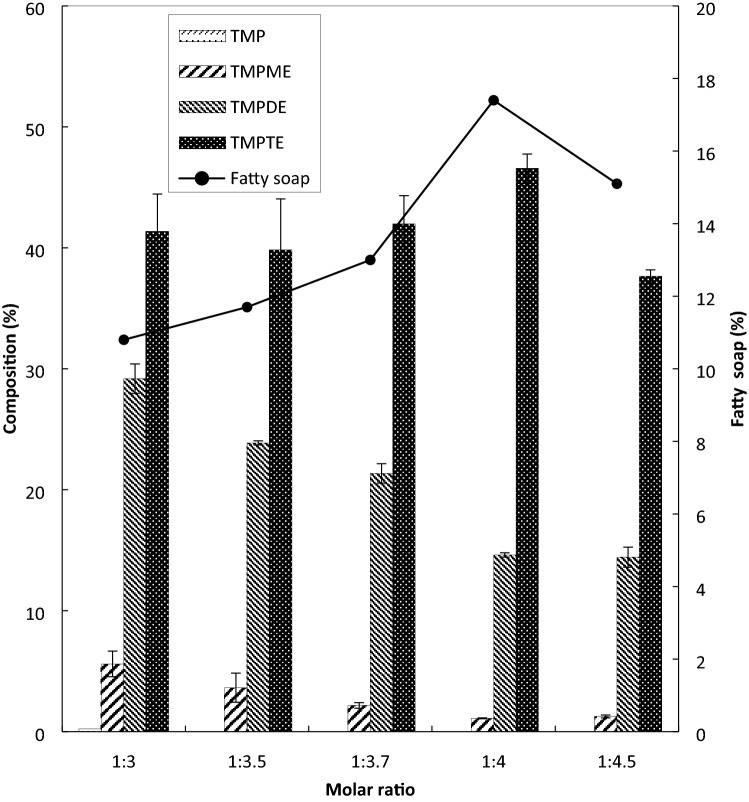
Effect of molar ratio on esters composition (at temperature 130 °C, pressure 10 mbar, catalyst 0.6 wt%, time 10 min).

The effect of molar ratio of TMP to PME is presented in Fig. 4. During the experimental works, the molar ratio of TMP to PME was manipulated at 1:3, 1:3.5, 1:3.7, 1:4, and 1:4.5. Other factors were maintained constant at 130 °C, 10 mbar, 0.6 wt.% catalyst and 10 min reaction time. The molar ratio of the reactants may determine the direction of the reaction. Excess PME was used to keep the reversible reaction intensifying to the right side, hence generating more products. Moreover, the removal of the by-product, in this case, methanol, via vacuum suction also helps to produce more TMPTE based on Le Chatelier’s principle. When the molar ratio of TMP to PME was set at 1:3, the TMPTE composition was high at 41.4 wt.%. However, the reaction was still incomplete based on the presence of traces of TMP in the product. The highest yield was obtained at molar ratio 1:4, with TMPTE, TMPDE, and TMPME being at 46.6, 14.6, and 1.1 wt.%, respectively. The standard deviation (SD) of each product, TMPTE, TMPDE, and TMPME are between 0.53–4.21, 0.17–1.22, and 0.04–1.20, respectively.

The reverse reaction steps became intense when higher molar ratios of TMP to PME were used. In addition, an excess volume of PME may dilute the catalyst, thus reducing the effective molecular interaction with fatty acid. This leads to a lower soap production at 15.1% for the molar ratio of TMP to PME at 1:4.5. Moreover, since PME has a higher value of dielectric constant than TMP, the microwave radiation is most probably absorbed by PME, leaving less amount of microwave energy to be absorbed by TMP. An adequate amount of microwave energy must be available for all reactants for the effective transesterification of PME and TMP.

### Effect of vacuum pressure

**Figure 5:**
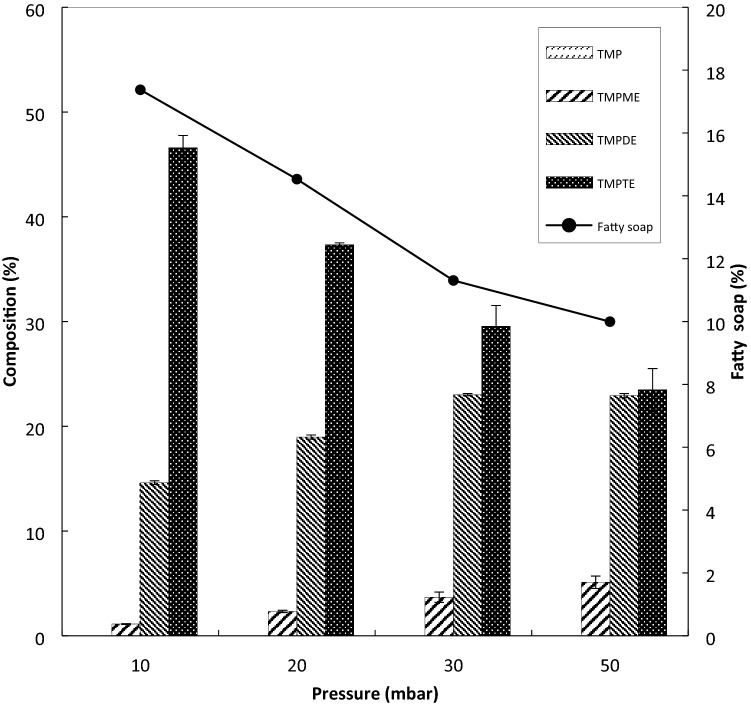
Effect of pressure on esters composition (at temperature 130 °C, catalyst 0.6 wt%, molar ratio 1:4, time 10 min).

The effect of pressure on the composition of TMP esters was investigated. The vacuum pressure was varied at under 10, 20, 30 and 50 mbar, while other factors were kept constant at 130 °C, 0.6 wt.% catalyst amount, reaction time of 10 min and molar ratio of TMP to PME at 1:4. Based on Fig. 5, it was observed that under low vacuum at 30 to 50 mbar, the process conditions were not enough to suppress the formation of partial esters, which are TMPME and TMPDE, to form PME. The standard deviation (SD) of each product, TMPME, TMPDE, and TMPTE are between 0.04–0.59, 0.11–0.20, and 0.21–2.05, respectively.

The present study suggests that when fatty acids from the hydrolysis of PME are reacted with sodium methoxide, fatty soap and methanol will be formed. Moreover, when the suction of methanol from the reactor is not strong enough, this condition may cause the accumulation of the methanol in the system. As a result, the interaction between fatty acids with sodium methoxide molecules to form fatty soap has been hindered. The minimum amount of fatty soap of 10% was obtained at 50 mbar, compared to 17% of fatty soap at 10 mbar. Nevertheless, the highest composition of TMPTE was observed at 10 mbar, despite the high formation of fatty soap. The result implies that the highest rates of TMPTE and fatty soap may have happened at 10 mbar, despite the competition between the transesterification and saponification reactions.

**Figure 6:**
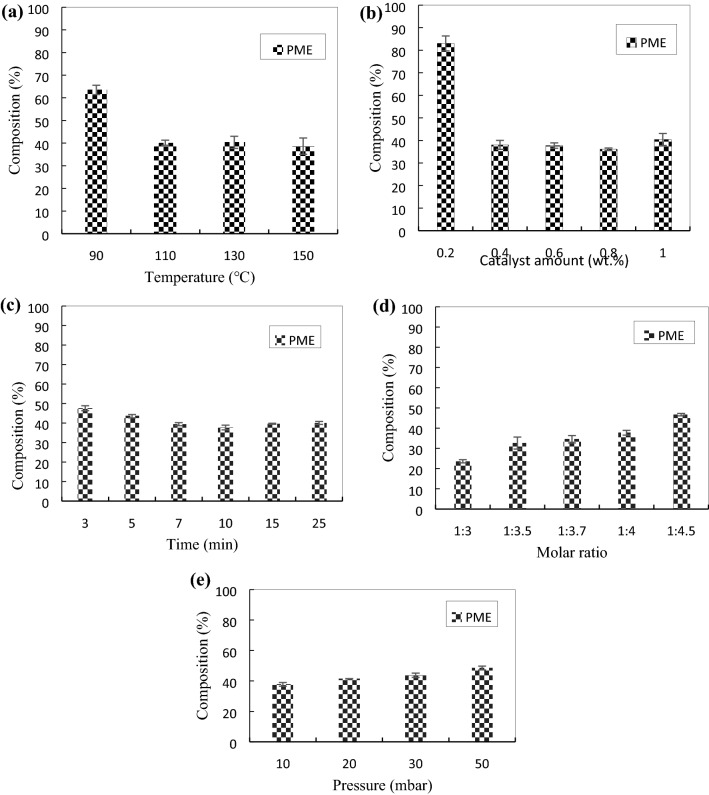
Composition of PME at different (**a**) temperature, (**b**) catalyst amount, (**c**) time, (**d**) molar ratio and (**e**) pressure.

Palm oil methyl ester (PME) is chosen as an excess reactant to drive the forward reaction to completion (producing more products). Figure 6a–e represents the composition of PME at a different temperature, catalyst amount, reaction time, molar ratio, and pressure. A range of standard deviation of PME data was between 0.10–3.61 which was relatively small. The molar ratio of PME to TMP was fixed at 4:1 for operating parameters such as temperature, catalyst amount, time, and pressure. Generally, the lowest composition of PME denotes that more PME is consumed in the transesterification, thus high yield of TMPTE is obtained. When the temperature was varied, while other factors were maintained, the highest PME at 63.5% was observed at 90 °C, as shown in Fig. 6a. The results showed that 90 °C is the least favourable reaction temperature due to the lowest conversion of the reactant. At 150 °C, the lowest PME composition may due to a vaporization at high temperature at vacuum pressure. As the reaction temperature increases, the more volatile PME especially having fatty acid chain of C12 and shorter may have escaped together with methanol in the vacuum line, hence decreases the overall composition of PME.

The trend of decrease in the composition of PME with temperature and catalyst amount is similar. As the amount of catalyst increases, more PME would be used in the reaction to produce the desired product TMP. Above 0.4 wt.% of catalyst, the PME composition remained almost unchanged. However, at 0.8 wt.% of catalyst, the composition of PME is slightly lower at 36.12% as in Fig. 6b. Yet, the contemplating economic issue contributes to the decision to select 0.6 wt.% of catalyst (37.74% of PME) is chosen as the best amount of catalyst. In Fig. 6c the effect of reaction time on PME is less pronounced. Extending the reaction time only affected the PME marginally. For the effect of molar ratio to PME composition, the PME composition is directly proportional to the molar ratio as shown in Fig. 6d. As the ratio increases, definitely more PME is present in the reaction media. At higher pressure, 50 mbar, more PME remained in the reaction because the reverse backward reaction could not be suppressed. At lower pressure,10 mbar, the forward reaction dominates the reversible reaction with the continuous removal of methanol, hence most PME reacted to form TMP esters as shown in Fig. 6e.

### Biolubricant base oil properties

In the current study, the microwave-assisted transesterification between PME and TMP has been successfully completed in 10 min to obtain TMPTE composition at 47 wt.%. After a fractionation process to remove the excess PME from the liquid product, the composition of TMPTE was 63 wt.%. The fractionated liquid product is also known as a biolubricant base oil. On the other hand, the conventional transesterification in the previous studies took 5–6 times longer for the reaction completion^14,42^. The properties of the base oil are tabulated in Table 2. The viscosities of the base oil at 40 and 100 °C in the current study were on a par with the results obtained by Hamid et al.^14^ and Yunus et al.^45^. However, the pour point obtained by the microwave-assisted reaction was − 18 °C, which is not as low as the pour points in the previous studies. The result is probably due to the high content of TMPDE in the final product, thus affecting the pour point value.

**Table 2:** Properties of TMP esters base oil.

Properties	Standard	TMP esters^a^	TMPTE^b^	TMPE^c^
TMPTE (wt%)	NA	63	95	98
Kinematic viscosity at 40 °C (cSt)	ASTM D445	45.3	45.7	48.4
Kinematic viscosity at 100 °C (cSt)	ASTM D445	9.0	9.5	9.9
Pour point (°C)	ASTM D 97	− 18	− 27	− 32

^a^TMP esters from current study.

^b^TMPTE from Hamid et al.^14^.

^c^TMPE from Yunus et al.^45^.

The efficiency of the microwave-assisted reaction was later compared to the conventional heating of the transesterification between PME and TMP. The operating conditions for both methods were at 130 °C, 10 min reaction time, vacuum pressure of 10 mbar, sodium methoxide catalyst at 0.6 wt.% and TMP to PME molar ratio of 1:4, with three replications. The composition of TMPTE for the conventional reaction at 10 min of reaction time only achieved 25.5 wt.%, followed by TMPDE and TMPME compositions at 16.3 and 2.1 wt.%, respectively. Conversely, a higher composition of TMPTE at 46.6 wt.% was obtained by using a microwave-assisted reaction, where the compositions of TMPDE and TMPME were recorded at 14.6 and 1.1 wt.%, respectively. Thus, it is to be inferred that the microwave-assisted reaction helps in the time saving and better productivity. Nonetheless, the fatty soap production from the conventional method was lower at 12%, compared to 17% fatty soap from microwave-assisted method. The result was due to the competitive main and side reactions as mentioned earlier.

Approximately 119 g of TMPE was produced at 130 °C as shown in Table 3. TMP which is hygroscopic has a high affinity towards moisture, approximately 1 wt.% from TMP^50^. Thus, PME could be hydrolysed in the presence of water to form free fatty acids (FFA). When FFA reacts with the alkaline catalyst, fatty soap will be formed. The catalyst, sodium methoxide was assumed to have negligible moisture content. Commercially, sodium metal is directly reacted to methanol to produce sodium methoxide. Moreover, the proper storage of the sodium methoxide usually does not contain any trace of moisture. The yield of the product depends on the conversion of the limiting reactant (TMP). At optimum conditions, the conversion of TMP was 100%. The enthalpy calculations are as follows:

4 $$Enthalpy for water and methanol = \smallint CpdT + Hf + \Delta Hv$$

5 $$Enthalpy for TMP = \Delta Hfusion + \smallint CpdT + Hf$$

6 $$Others: Total enthalpy = \smallint CpdT + Hf$$

*CpdT* is the integration of heat capacity of material at constant pressure, at set and reference temperature, 25 and 130 °C, respectively. *H*_*f*_ is the heat of formation, *ΔH*_*v*_ is heat of vaporization, and $\Delta Hfusion$ is heat of fusion.

**Table 3:** Energy balances of microwave-assisted and conventional transesterification.

Stream	Input			Microwave-assisted reaction output			Conventional reaction output		
	Mass (g)	Mol	∑n_in_H_in_ (kJ)	Mass (g)	Mol	∑n_out_H_out_ (kJ)	Mass (g)	Mol	∑n_out_H_out_ (kJ)
PME	80	0.27128	− 147.86	59.13	0.20051	− 109.29	59.13	0.20051	− 109.29
TMP	9.10	0.06780	− 83.50	0	0	0.00	0	0	0.00
Sodium Methoxide	0.16	0.00297	− 1.20	0	0	0.00	0	0	0.00
Water	0.09	0.00505	− 0.56	0	0	0.00	0	0	0.00
TMPME	0	0	0.00	0.99	0.00249	− 2.28	2.42	0.00609	− 5.57
TMPDE	0	0	0.00	13.13	0.01990	− 23.84	19.23	0.02914	− 34.91
TMPTE	0	0	0.00	41.91	0.04541	− 70.62	30.06	0.03257	− 50.65
Methanol	0.37	0.01168	0.00	2.74	0.08542	− 1.18	2.74	0.08542	− 1.18
Fatty acids	0	0	0.00	0.58	0.00208	− 1.57	0.58	0.00208	− 1.57
Fatty soap	0	0	− 1.96	0.90	0.00297	− 14.33	0.90	0.00297	− 14.33
Total	89.72	0.35878	− 235.07	119.37	0.35878	− 223.11	115.05	0.35878	− 217.51
						ΔH (kJ) = 11.97		ΔH (kJ) = 17.57	

Microwave heating is significantly more effective than the conventional heating. The total energy consumption during the transesterification between PME and TMP was calculated as follows in Eq. (8):

7 $$\Delta \dot{H}{ } = n_{Out} \dot{H}_{Out} - n_{In} \dot{H}_{In}$$

8 $$Total energy consumption = E_{Heat} + E_{Vacuum} - \Delta \dot{H}.$$

where *E*_*Heat*_ is the energy consumption by heating (kJ), *E*_*Vacuum*_ is the energy consumed by the vacuum pump (kJ), and $\Delta \dot{H}$ is energy absorbed by reaction system. The yield of TMPTE was calculated as in Eq. (9):

9 $$Yield of TMPTE=\frac{Experimental mass of TMPTE}{Theoretical mass of TMPTE}\times 100\%$$

Since the transesterification of PME and TMP is an endothermic reaction, energy is needed to be absorbed by the reaction. The values of energy absorbed by microwave-assisted and conventional reactions were 11.97 kJ and 17.57 kJ, respectively. As shown in Table 3, it was found that microwave-assisted reaction for 10 min resulted in a higher TMPTE yield (67.0%). Less energy consumption (1195.0 kJ) was achieved as compared to the conventional reaction system. In Table 4, the yield of TMPTE obtained by using conventional heating was only 48.1 wt.% at 3786.4 kJ of energy consumption. For both heating methods, the inlet enthalpy was higher than the outlet enthalpy. The yield of the microwave-assisted reaction was high. As a result, high outlet enthalpy was released from the microwave system. Based on Eq. (7), the energy absorbed by the reaction was lower than energy absorbed by the conventional reaction. Moreover, the energy released from the conventional heating was 3.6-fold higher. Hence, the microwave-assisted reactor system gave a total energy saving of 68.4%.

**Table 4:** Comparison of trimethylolpropane triesters (TMPTE) yields between the conventional heating and microwave system at 10 min of transesterification.

Method	Preheating and reaction time (min)	Energy consumption of preheat and reaction heating (kJ)	Energy consumption of vacuum pump (kJ) (10 min)	Energy absorbed by reaction system (kJ)	Total energy consumption (kJ)	Yield of TMPTE (wt.%)
Conventional heating	40	3588	216	17.57	3786.4	48.1
Microwave system	13	990.6	216	11.97	1194.6	67.0

Although the reaction time of both reactions was set for 10 min, the preheating time to heat the liquid reaction mixture was different for both heating systems. For the microwave system, the required pre-heating time was only 3 min, while 30 min was needed for the conventional heating. The ten-fold increase in pre-heating time of the conventional heating, compared to the microwave heating, has caused a significantly higher heating energy consumption, as shown in Table 4. The total energy consumption result for both heating methods implies that an appropriate use of the microwave system helps a lot in energy saving. Similar results were also reported in previous research regarding the reduction of the energy consumption using the microwave-assisted reactions^51–53^.

Our previous study^44^ on the kinetics and thermodynamics investigation found that the forward-reaction activation energy of the microwave-assisted transesterification of TMP and PME was approximately 54.2–72.6 kJ/mol. Interestingly, the rates of forward reaction were dominated and faster than the reverse reactions. The reverse reaction consumed more time and energy with activation energy 70.7–99.9 kJ/mol. These findings are within the range of oil transesterification reactions using a base catalyst^54^. The second-order reversible reaction kinetics proposed solved the reaction rate constants using MATLAB simulation—Ordinary Differential Equation (ODE) function. Corresponding to the current study, the activation energy indicated a rapid reaction induced by microwave heating. As a result, the conversion of TMP and PME to monoester, diester, and triester has taken place step by step very quickly. The production of high TMPTE was at high temperature (130 °C), and the reaction rate constants which are highly temperature-dependent improved significantly at an optimum temperature.

### Fourier transform infra-red for TMP esters

**Figure 7:**
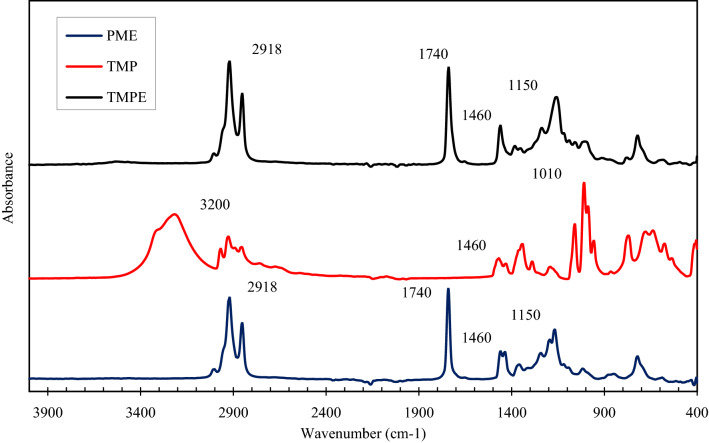
FTIR spectra for TMP, PME and TMP ester.

The FT-IR spectra of the raw materials used (TMP and PME) in the transesterification reaction were analysed and compared to the product (TMP esters) obtained is presented in the Fig. 7.

TMP demonstrated a distinct broad peak from 3500–3000 cm^−1^ region that refers to the stretching of the alcohol O–H group. The intense peak at 1010 cm^−1^ indicates the stretching of C–O bonding by an alcohol group. The peaks at 3200 and 1010 cm^−1^ denotes that C–O and O–H group did not emerged in TMPE because the bonds were broken when attacked by sodium methoxide ions to produce alkoxides and protonated catalyst, as shown in the previous studies^44^. As a result, the O–H bond owned by TMP has been replaced with methyl ester. PME and TMPE posed the same peak at region 2918 and 1740 cm^−1^. These wavelengths belong to alkane by its C–H bond and the C=O bond of the carbonyl group. The same ester trend was also reported in the previous study of various saturation level of palm oil^55^. Moreover, Rabelo et al.^56^ also obtained the carbonyl group at 1742 cm^−1^ during the analysis of soybean oil-based methyl ester. In addition, the ester carbonyl functional group was also reported at 1743 cm^−1^ ^57^. As the main back bone of the ester, TMP esters display the most similar plot with the PME at which 1150 cm^−1^ refers to the C–O stretching for ester (1210–1163 cm^−1^). The factor of the current result may also be contributed to by the excess of PME used in the reaction. PME, TMP and TMP esters showed an intense peak at 1460 cm^−1^ that denotes the C–H bending for alkanes (1465–1380 cm^−1^).

### Thermogravimetric (TG) and differential scanning calorimetric (DSC) analysis

**Figure 8:**
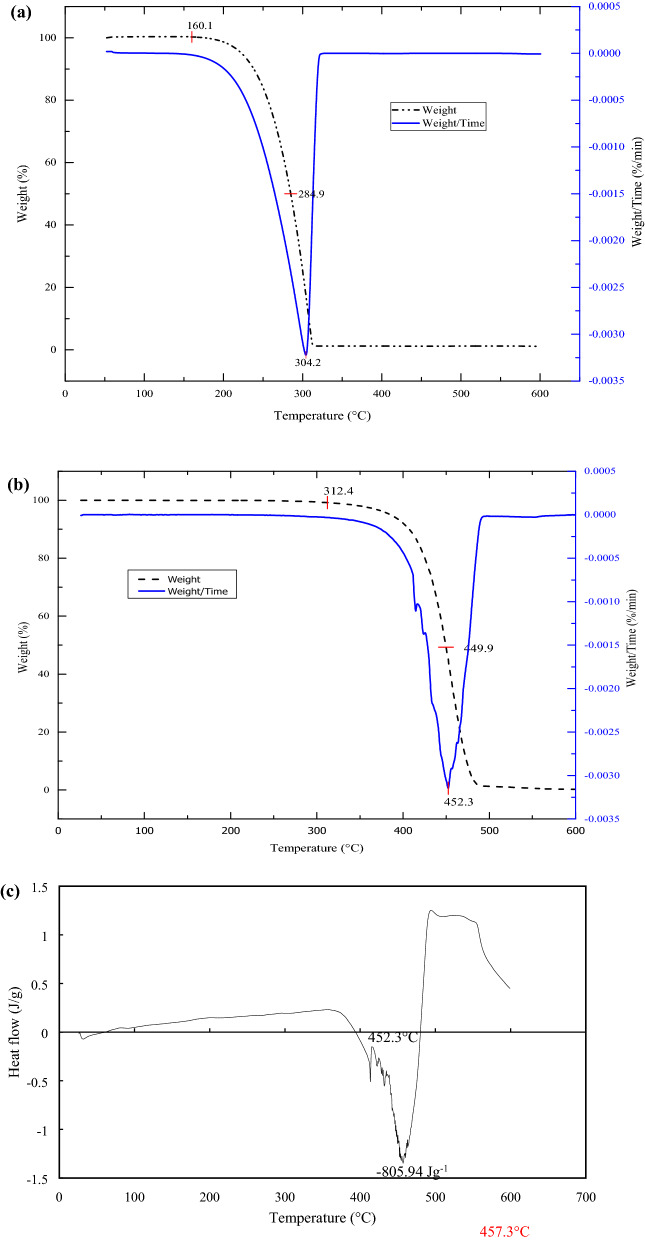
(**a**) TGA and DTG curve of palm oil methyl ester (PME) (**b**) TG and DTG curve of trimethylolpropane esters (TMPE) (**c**) DSC curve for trimethylolpropane triester (TMPE).

A common tool for thermal analysis, especially thermal stability, is TGA. The analysis is widely used in the chemical engineering field for oils and polymer constituents^58^. Three thermograms that are commonly discussed: TGA curve, DTG or derivative of TGA curve, and DSC curve which represent the heat flow of the sample, as illustrated in Fig. 8a,b, respectively. The TGA curve of weight loss of PME (the reactant) and TMPE (the final product) against the temperature in Fig. 8a,b showed that the starting temperature for degradation or reduction of PME and TMPTE weights took place at 170 and 312 °C, respectively. At 284 °C, almost 50% of PME was decomposed. Most PME was decomposed at 304 °C. It was found that 50% of TMP ester’s sample was decomposed at a temperature of 449.9 °C. The initial weight loss was due to the methyl ester (mainly methyl oleate and palmitate) combustion or evaporation. The DTG curve showed that the temperature range of material combustion with 452.3 °C was the temperature most material was decomposed. These results showed that TMP esters have good thermal stability and are ready for various base derivative purposes, such as in lubrication or metal working. A quite similar result was reported^59^, with the onset temperature of high oleic TMP esters at 435 °C, which was conducted under conventional heating. The sample weight was also reduced to half at 488 °C. Moreover, the higher temperature resistance obtained by the study was due to the employment of a high oleic composition of PME. The thermal breakdown of methyl ester consists of volatile lighter hydrocarbon constituents, carbon dioxide, and carbon monoxide^60^. The DSC curve in Fig. 8c displays an endothermic characteristic attributed to the volatilisation of methyl ester. Furthermore, the energy consumed by the sample during the phase transformation was − 805.94 Jg^−1^.

### ^1^H-NMR for synthesis biolubricant

**Figure 9:**
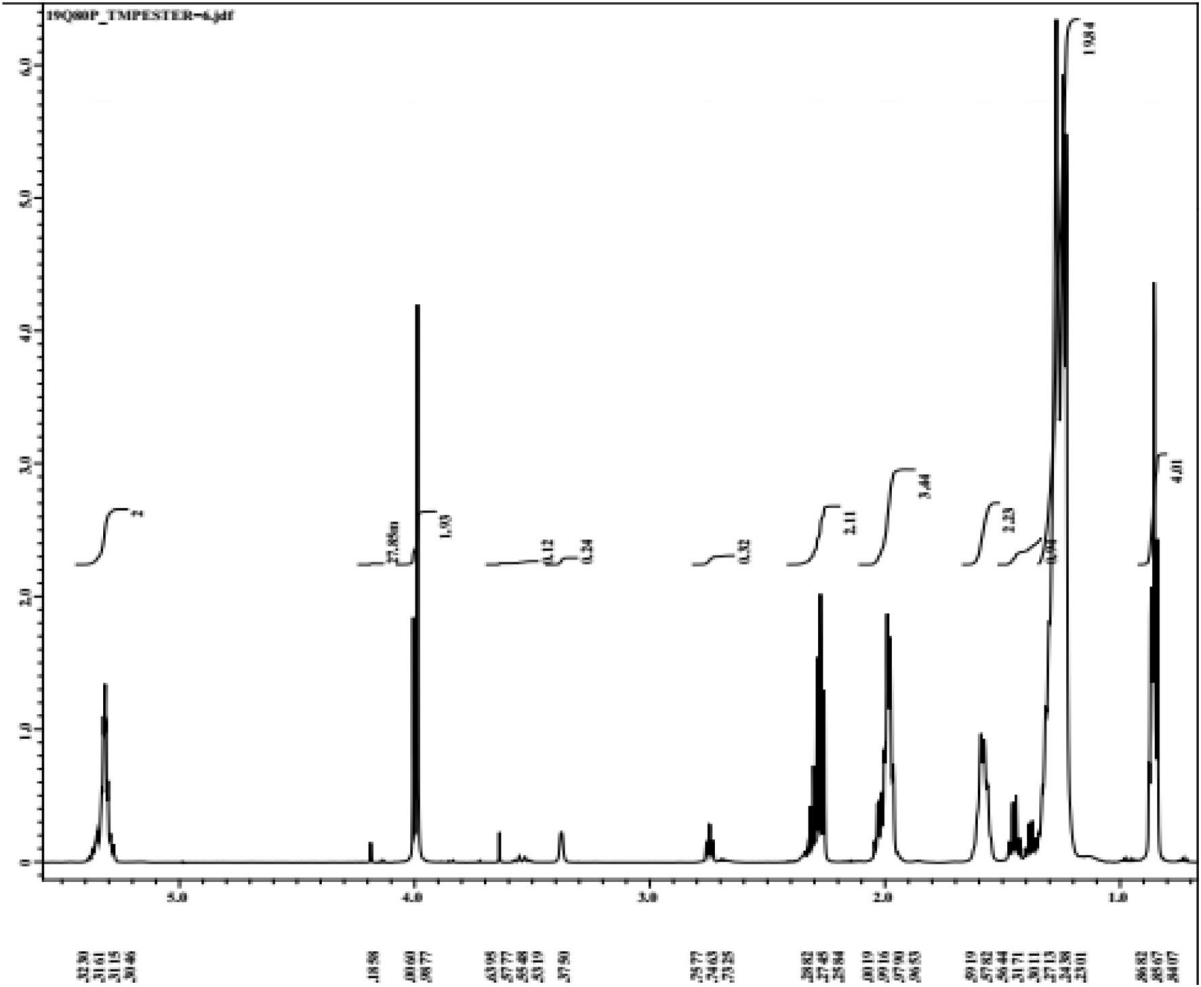
^1^H-NMR for TMP esters.

^1^H-NMR is a valuable technique for quality evaluation and quantification of methyl ester. A typical ^1^H-NMR spectrum of TMP (Fig. 9) shows peaks of aliphatic hydrogens at 5.32 ppm and 3.6–0.84 ppm Methyl TMP. Figure 9 shows a singlet near 3.63 ppm, related to the methoxy group, which allows TMP quantification of TMP synthesis, and a triplet of α-CH_2_ protons at 2.27 ppm. These two peaks are the distinct peaks for the confirmation of methyl esters present in TMP. Other observed peaks were at 0.84, 0.85 and 0.86 ppm of terminal methyl protons. Another signal at 1.9 ppm is related to methylene protons of the carbon chain, a signal at 1.31 ppm from the β**-**carbonyl methylene protons, and at 5.31 ppm due to olefinic hydrogen. TMP production can also be followed through the decrease of peaks at 4.18–3.98 ppm, which are assigned to hydrogens of O–CH_3_ moiety from the palm oil methyl ester (PME). Hence, these characteristic peaks from methyl TMP and PME can be used for their quantification in the transesterification reaction. Thus, the goal of the present work was to obtain the optimum condition for TMPTE preparation from palm oil methyl ester (PME).

## Conclusion

The microwave-PWM assisted transesterification of PME and TMP has successfully reduced the time of reaction compared to the conventional reaction system. In 10 min, the composition of TMPTE at 63 wt.% was achieved at 130 °C, 0.6 wt.% sodium methoxide, molar ratio TMP: PME of 1:4, and under 10 mbar. While it took 1 hr for the conventional method to achieved similar conversion. However, microwave heating system not only accelerates the transesterification reaction, but also other competing reactions, namely the hydrolysis and saponification reactions. The energy consumption by using the fabricated microwave system was greatly reduced compared to the conventional heating method. However, further investigation on the kinetic study is necessary for better understanding of the coupled electromagnetic and heat transfer for the transesterification of PME and TMP.

## Materials and methods

### Materials

The polyol, trimethylolpropane (TMP) was obtained from Acros Organics—Thermo Fisher Scientific (Malaysia) and the palm oil methyl ester was purchased from Carotino Sdn. Bhd. (Malaysia). Sodium methoxide was obtained from Sigma Aldrich Chemicals Co (Malaysia), while both ethyl acetate and N, O-Bis(trimethylsilyl)trifluoroacetamide (BSTFA) were purchased from Fluka Chemie AG (Switzerland). Palm oil methyl ester (PME) used in the study consisted of a collection of fatty acid esters with various molecular weights (mainly oleic). This complex nature of plant-based methyl ester may generate a diversity of products from multiple reactions. An assumption was made such that PME was considered as a single component (C:18), based on its major constituent (approximately 75% w/w) in the compound, in order to simplify the reaction model. The details of the components are presented in the Table 5.

**Table 5:** Methyl ester composition of PME . (Adapted from Carotino Sdn. Bhd., 2012).

Methyl ester	Composition (% w/w)
Methyl myristate (C14:0)	0.4
Methyl palmitate (C16:0)	3.7
Methyl stearate (C18:0)	3.6
Methyl oleate (C18:1)	74.9
Methyl linoleate (C18:2)	17.4

### Experimental design

In this experimental design, the one-variable-at-a-time (OVAT) technique is a general optimisation approach that involves the adjustment of one parameter at a time in order to elicit a response as a function of that one parameter. A number of researchers have verified and promoted the OVAT approach for optimising the transesterification reaction by examining parameters such as temperature, amount of catalyst, time of reaction, molar ratio, and pressure, which were varied as shown in Table 6. Each run was replicated three times with the average value, and the standard deviation of the best three readings, being evaluated and plotted in the graph. The results are compared, both qualitatively and quantitatively.

**Table 6:** Design of experiments for microwave-assisted transesterification for PME and TMP.

Experimental objectives	Parameters				
	Temperature (˚C)	Catalyst amount (wt%)	Reaction time (min)	Molar ratio (TMP: PME)	Pressure (mbar)
Effect of temperature	90, 110, 130,150	1.0	10	1:4	10
Effect of catalyst amount	130	0.2, 0.4, 0.6, 0.8, 1.0	10	1:4	10
Effect of reaction time	130	0.6	3, 7, 10, 15, 25	1:4	10
Effect of molar ratio TMP to PME	130	0.6	10	1:3, 1:3.5, 1:3.7, 1:4, 1:4.5	10
Effect of pressure	130	0.6	10	1:4	10, 20, 30, 50

### Microwave-assisted transesterification reaction

A microwave-PMW Dixson 2301 (Dixson FA Engineering, Sdn., Bhd., Shah Alam, Selangor, Malaysia) operated at 2.45 GHz was fabricated for biodiesel production^34^. This system controls the operating temperature inside the microwave cavity and prevent the overheating of materials which could damage the structure of methyl ester. Besides that, Lokman et al.^34^ reported that the improvement of esterification reaction by the repetition of the rapid strong microwave energy to control the temperature, surpassing the normal continuous microwave at the same energy level at reduced time.

The transesterification of PME and TMP was conducted in a 250 ml three-neck flask under vacuum conditions with the mass of PME kept constant in all experiments. PME was dried overnight in the oven at 105 °C prior to experiments to reduce the moisture content. Subsequently, 80 g of PME and the appropriate amount of TMP were mixed in the three-neck flask equipped with a thermocouple and a sample port and placed in the centre of the microwave oven. The setup was connected to a vacuum pump. The mixture was stirred by a magnetic stirrer throughout the reaction. Each run was heated by using microwave heating for 3 min before the addition of the catalyst, to provide uniform mixing at the set temperature. Then, a small amount of catalyst, which was calculated based on the weight of reactant, was poured into the hot mixture, and the microwave heating continued as per the set time. Vacuum pressure was controlled gradually to the prescribed set point to avoid a sudden burst or spillage of reactant. The removal of air from the system also eliminated the by-product (methanol). Methanol, the by-product must be continuously removed to drive the forward reaction and obtain a higher yield of TMP esters.

After the reaction reached completion for the targeted reaction time, a drop of sample was taken out by using a syringe and was prepared for a gas chromatography analysis. The dielectric constant, ε’, and loss factor, ε”, for each reactant, was measured at room temperature by an open-ended coaxial probe (HP 85070B, Agilent, Palo Alto, CA, USA) that was connected to a network analyser (HP 8753C, Agilent, Palo Alto, CA, USA) at a frequency in the range of 1–4 GHz. A high dielectric constant value defines the ability of a material to absorb microwave energy, while a high loss factor denotes the capability of the material for rapid heat transfer^43,61^.

### Fatty soap separation

After the reaction was completed, the product mixture was allowed to cool down to room temperature, prior to separating the fatty soap by-product from the reaction mixture. The Whatman qualitative filter paper no. 5 were used for the separation process. The mass of soap was calculated by using Eq. (10):

10 $$Fatty soap= \frac{Mass of solid material (g)}{Total mass of reactant (g)} \times 100\%$$

### Removal of excess palm oil methyl ester (PME)

In the transesterification reaction, TMP is the limiting reactant while PME is the excess reactant. Since there was an excess of PME used, this was removed at the end of the process, to obtain a high purity of TMP esters before the analysis was conducted. A hotplate was set at temperature of 260 °C (to achieve temperature 180 °C inside the reactor) to heat up the reaction mixture in a three-neck flask that was immersed in a silicone oil bath. The three-neck flask, with sampling port equipped with a thermometer, was connected to a relief valve and a condenser with a water supply at room temperature. An Erlenmeyer flask, which was connected to a vacuum pump, was used to collect the excess of PME. The relief valve was gradually closed to introduce low pressure of at least 1.4 × 10^–2^ mbar for 4 h.

### Material and energy balance of transesterification reaction of palm oil methyl ester (PME) and trimethylolpropane (TMP)

Based on Eq. (1)–(3) for transesterification reaction of PME and TMP, the hydrolysis and saponification reaction were assumed as follow:

11 $${\text{PME }} + {\text{ H}}_{{2}} {\text{O}} \to {\text{Free fatty acid }}\left( {{\text{FFA}}} \right) \, + {\text{ CH}}_{{3}} {\text{OH }}\left( {{\text{Methanol}}} \right)$$

12 $${\text{FFA }} + {\text{ NaOCH}}_{{3}} \left( {\text{Sodium Methoxide}} \right) \to {\text{RCOONa }}\left( {{\text{FFAONa}}} \right) \, + {\text{ CH}}_{{3}} {\text{OH}}$$

The PME was hydrolyzed with moisture that presents in the PME and TMP thus producing FFA and methanol. FFA further reacted with the catalyst and formed fatty soap (FFAONa) and methanol. Several assumptions for the calculation are:

- The conversion of H_2_O to FFA is 100%
- No hydrolysis of TMPTE and TMPDE during transesterification
- There is no moisture in catalyst
- The composition of fatty acids, fatty soap, TMPME, TMPDE, and TMPTE were based on the composition of PME (Table 5)

### Characterisation of trimethylolpropane triesters

- Sample preparation
  A small drop of sample (0.01 ± 0.005 g) was diluted with 1.0 mL of ethyl acetate in a 2 mL vial and whirled thoroughly. Next, 0.5 mL of BSTFA was added and the mixture was swirled and then heated in a bath at temperature 40 to 50 °C for 10 min before the sample was injected into the gas chromatography—flame ionisation detector (GC-FID) system.
- GC-FID analysis
  GC-FID Perkin Elmer was used to analyse the ester using a specialised column of DB-5HT with 15 m $\times$ 0.32 mm $\times$ i.d. 0.1 µm. The methodology of GC analysis was established by Yunus et al.^62^, with some modifications in order to save time and gas consumption. The sample was injected into the system at 80 °C initial oven temperature for 3 min. The system was programmed to ramp at 6 °C /min up to 360 °C, and finally held at this condition for another 5 min. Hydrogen was used as a carrier gas with a flow rate of 26.67 mL/min, while nitrogen was consumed as a make-up gas. Only 0.1 µL of the sample was injected into the GC system and it took 27 min to analyse the sample.
- Fourier transform infra-red (FTIR) spectroscopy analysis
  The FTIR spectra of TMPTE was recorded using a Perkin Elmer 1650 spectrometer (USA). All the corresponding spectra were using an absorption mode with a wavelength range of 4000 to 500 cm^−1^.
- Thermogravimetric analysis (TGA) and differential scanning calorimetric (DSC) analysis
  The thermal characterisation of TMPTE by using TGA and DSC were carried out in air medium for the temperature range of 25 to 600 °C, at 50 ml/min. The heating rate was set at 10 °C/min. The equipment was from Mettler Toledo TGA/DSC 823 IHT (Columbus, USA).
- Nuclear Magnetic Resonance (NMR) Spectroscopy

Further determination of the characterising was conducted to determine the quality of TMPTE and quantification of methyl ester by ^1^H-NMR spectroscopy. The analysis was performed on JOEL NMR (model ECX500, USA) with chloroform as solvent operating at 500 MHz.

## References

[1] John, J. Global Lubricants Market Will Reach USD 146.3 Billion by 2024: Zion Market Research. *GlobeNewswire* https://www.globenewswire.com/news-release/2018/05/23/1510519/0/en/Global-Lubricants-Market-Will-Reach-USD-146-3-Billion-by-2024-Zion-Market-Research.html (2018).

[2] Peixoto C (2018). Chemical modification of Tilapia oil for biolubricant applications. J. Clean. Prod..

[3] Jering A (2010). Use of renewable raw materials with special emphasis on chemical industry. Eur. Top. Cent. Sustain. Consum. Prod..

[4] Panchal TM, Patel A, Chauhan DD, Thomas M, Patel JV (2017). A methodological review on bio-lubricants from vegetable oil based resources. Renew. Sustain. Energy Rev..

[5] Singh Y, Farooq A, Raza A, Mahmood MA, Jain S (2017). Sustainability of a non-edible vegetable oil based bio-lubricant for automotive applications : A review. Process Saf. Environ. Prot..

[6] Åkerman CO, Gaber Y, Ghani NA, Lämsä M, Hatti-Kaul R (2011). Clean synthesis of biolubricants for low temperature applications using heterogeneous catalysts. J. Mol. Catal. B Enzym..

[7] Nagendramma P, Kaul S (2012). Development of ecofriendly / biodegradable lubricants: An overview. Renew. Sustain. Energy Rev..

[8] Padmaja KV (2012). 10-Undecenoic acid-based polyol esters as potential lubricant base stocks. Ind. Crops Prod..

[9] Srivastava A (2013). Vegetable oils as lubestocks: A review. Afr. J. Biotechnol..

[10] Wagner H, Luther R, Mang T (2001). Lubricant base fluids based on renewable raw materials their catalytic manufacture and modification. Appl. Catal..

[11] Salimon J, Salih N, Yousif E (2012). Industrial development and applications of plant oils and their biobased oleochemicals. Arab. J. Chem..

[12] Gryglewicz S, Muszynski M, Nowicki J (2013). Enzymatic synthesis of rapeseed oil-based lubricants. Ind. Crop Prod..

[13] Aziz NAM, Yunus R, Rashid U, Muhammad A (2014). Application of response surface methodology (RSM) for optimizing the palm-based pentaerythritol ester synthesis. Ind. Crops Prod..

[14] Hamid HA (2016). Synthesis of high oleic palm oil-based trimethylolpropane esters in a vacuum operated pulsed loop reactor. Fuel.

[15] Kodali DR (2002). High performance ester lubricants from natural oils. Ind. Lubr. Tribol..

[16] Salimon J, Salih N, Yousif E (2010). Biolubricants: Raw materials, chemical modifications and environmental benefits. Eur. J. Lipid Sci. Technol..

[17] Mobarak HM (2014). The prospects of biolubricants as alternatives in automotive applications. Renew. Sustain. Energy Rev..

[18] Raof NA, Rashid U, Yunus R, Azis N, Yaakub Z (2016). Development of palm-based neopentyl glycol diester as dielectric fluid and its thermal aging performance. IEEE Trans. Dielectr. Electr. Insul..

[19] Worschech, K. (Loxstedt) *et al.* Stabilizer for Chlorine-containing Olefin Polymers, a Process for its Production and Polymers Containing the Stabilizer. (1991) doi:10.1016/j.(73).

[20] Yaakob Z, Mohammad M, Alherbawi M, Alam Z, Sopian K (2013). Overview of the production of biodiesel from waste cooking oil. Renew. Sustain. Energy Rev..

[21] Leung DYC, Wu X, Leung MKH (2010). A review on biodiesel production using catalyzed transesterification. Appl. Energy.

[22] Teixeira CB, Madeira Junior JV, Macedo GA (2014). Biocatalysis combined with physical technologies for development of a green biodiesel process. Renew. Sustain. Energy Rev..

[23] Trivedi J, Aila M, Sharma CD, Gupta P, Kaul S (2015). Clean synthesis of biolubricant range esters using novel liquid lipase enzyme in solvent free medium. Springerplus.

[24] Kamil RNM, Yusup S, Rashid U (2011). Optimization of polyol ester production by transesterification of Jatropha-based methyl ester with trimethylolpropane using Taguchi design of experiment. Fuel.

[25] Zhang S (2010). Rapid microwave-assisted transesterification of yellow horn oil to biodiesel using a heteropolyacid solid catalyst. Bioresour. Technol..

[26] Chang T-S (2012). Activity of calcium methoxide catalyst for synthesis of high oleic palm oil based trimethylolpropane triesters as lubricant base stock. Ind. Eng. Chem. Res..

[27] Hamid HA, Yunus R, Rashid U, Choong TSY, Al-Muhtaseb AH (2012). Synthesis of palm oil-based trimethylolpropane ester as potential biolubricant: Chemical kinetics modeling. Chem. Eng. J..

[28] Mazo P, Rios L, Estenoz D, Sponton M (2012). Self-esterification of partially maleated castor oil using conventional and microwave heating. Chem. Eng. J..

[29] Gunawan ER, Basri M, Rahman MBA, Salleh AB, Rahman RNZA (2005). Study on response surface methodology (RSM) of lipase-catalyzed synthesis of palm-based wax ester. Enzyme Microb. Technol..

[30] Liu K-S (1994). Preparation of fatty acid methyl esters for gas-chromatographic analysis of lipids in biological materials. J. Am. Oil Chem. Soc..

[31] Hamid HA (2018). Synthesis study of high oleic palm oil-based trimethylolpropane triesters: Response surface methodology based optimization. Chiang Mai J. Sci..

[32] Ivan-Tan CT, Islam A, Yunus R, Taufiq-Yap YH (2017). Screening of solid base catalysts on palm oil based biolubricant synthesis. J. Clean. Prod..

[33] Kusuma HS, Ansori A, Wibowo S, Bhuana DS, Mahfud M (2018). Optimization of transesterification process of biodiesel from Nyamplung (*Calophyllum inophyllum* Linn) using microwave with CaO catalyst optimization of transesterification process of biodiesel from Nyamplung (*Calophyllum inophyllum* Linn) using microwa. Korean Chem. Eng. Res.

[34] Lokman IM, Rashid U, Taufiq-Yap YH (2015). Microwave-assisted methyl ester production from palm fatty acid distillate over a heterogeneous carbon-based solid acid catalyst. Chem. Eng. Technol..

[35] Tippayawong N, Sittisun P (2012). Continuous-flow transesterification of crude jatropha oil with microwave irradiation. Sci. Iran..

[36] Campos DC, Dall Oglio EL, de Sousa PT, Vasconcelos LG, Kuhnen CA (2014). Investigation of dielectric properties of the reaction mixture during the acid-catalyzed transesterification of Brazil nut oil for biodiesel production. Fuel.

[37] Gude VG, Patil P, Martinez-guerra E, Deng S (2013). Microwave energy potential for biodiesel production. Sustain. Chem. Process..

[38] Tan SX, Lim S, Ong HC, Pang YL (2019). State of the art review on development of ultrasound-assisted catalytic transesterification process for biodiesel production. Fuel.

[39] Lieu T, Yusup S, Moniruzzaman M (2016). Kinetic study on microwave-assisted esterification of free fatty acids derived from *Ceiba pentandra* Seed Oil. Bioresour. Technol..

[40] Nayak SN, Bhasin CP, Nayak MG (2019). A review on microwave-assisted transesterification processes using various catalytic and non-catalytic systems. Renew. Energy.

[41] Komarov VV, Tang J (2004). Dielectric permittivity and loss factor of tap water at 915 MHz. Microw. Opt. Technol. Lett..

[42] Yunus R, Idris A (2003). Development of optimum synthesis method for transesterification of palm oil methyl esters and trimethylolpropane to environmentally acceptable palm oil-based lubricant. J. Oil Palm Res..

[43] Lidstrom P, Tierney JP, Wathey B, Westman J (2005). Microwave assisted organic synthesis—a review. Int. J. Chem..

[44] Aziz, N. A. M. *et al.* Kinetics and thermodynamics of synthesis of palm oil-based trimethylolpropane triester using microwave irradiation. *J. Saudi Chem. Soc.* In Press, (2020).

[45] Yunus R, Fakhru A, Ooi TL, Omar R, Idris A (2005). Synthesis of palm oil based trimethylolpropane esters with improved pour points. Ind. Eng. Chem. Res..

[46] Lin YC, Hsu KH, Lin JF (2014). Rapid palm-biodiesel production assisted by a microwave system and sodium methoxide catalyst. Fuel.

[47] Azcan N, Yilmaz O (2013). Microwave assisted transesterification of waste frying oil and concentrate methyl ester content of biodiesel by molecular distillation. Fuel.

[48] Terigar BG, Balasubramanian S, Lima M, Boldor D (2010). Transesterification of soybean and rice bran oil with ethanol in a continuous-flow microwave-assisted system: Yields, quality, and reaction kinetics. Energy Fuels.

[49] Wu X, Ying P, Liu J (2009). An International Journal for rapid communication of synthetic organic chemistry lithium chloride—assisted selective hydrolysis of methyl esters under microwave irradiation. Synth. Commun..

[50] Chang T (2015). Synthesis of High Oleic Palm Oil-Based Trimethylolpropane Esters Using Calcium Methoxide Catalyst for Use as Lubricant Base Stock.

[51] Dehghan L, Golmakani MT, Hosseini SMH (2019). Optimization of microwave-assisted accelerated transesterification of inedible olive oil for biodiesel production. Renew. Energy.

[52] Ding H (2018). Process intensification of transesterification for biodiesel production from palm oil: Microwave irradiation on transesterification reaction catalyzed by acidic imidazolium ionic liquids. Energy.

[53] Teng WK, Ngoh GC, Yusoff R, Aroua MK (2016). Microwave-assisted transesterification of industrial grade crude glycerol for the production of glycerol carbonate. Chem. Eng. J..

[54] Moradi GR (2015). Kinetic comparison of two basic heterogenous catalysts obtained from sustainable resources for transesterification of waste cooking oil. Biofuel Res. J..

[55] Sherazi STH, Talpur MY, Mahesar SA, Kandhro AA, Arain S (2009). Main fatty acid classes in vegetable oils by SB-ATR-Fourier transform infrared (FTIR) spectroscopy. Talanta.

[56] Rabelo SN, Ferraz VP, Oliveira LS, Franca AS (2015). FTIR analysis for quantification of fatty acid methyl esters in biodiesel produced by microwave-assisted transesterification. Int. J. Environ. Sci. Dev..

[57] Rohman A, Man YBC (2010). Fourier transform infrared (FTIR) spectroscopy for analysis of extra virgin olive oil adulterated with palm oil. Food Res. Int..

[58] Jain S, Sharma MP (2012). Application of thermogravimetric analysis for thermal stability of Jatropha curcas biodiesel. Fuel.

[59] Raof, N. A., Yunus, R., Rashid, U. & Azis, N. Effect of molecular structure on oxidative degradation of polyol ester. in *Proceedings of Asia International Conference on Tribology* 353–354 (2018).

[60] Borugadda VB, Goud VV (2014). Thermal, oxidative and low temperature properties of methyl esters prepared from oils of different fatty acids composition: A comparative study. Thermochim. Acta.

[61] Thostenson ET, Chou T (1999). Microwave processing : fundamentals and applications. Compos. Part A Appl. Sci. Manuf..

[62] Yunus R, Lye OT, Fakhrul-Razi A, Basri S (2002). A simple capillary column GC method for analysis of palm oil-based polyol esters. J. Am. Oil Chem. Soc..

